# chromploid: An R package for chromosome number evolution across the plant tree of life

**DOI:** 10.1002/aps3.1037

**Published:** 2018-04-11

**Authors:** Rosana Zenil‐Ferguson, J. Gordon Burleigh, José Miguel Ponciano

**Affiliations:** ^1^ Department of Biological Science University of Idaho Moscow Idaho 83844 USA; ^2^ Department of Ecology, Evolution, and Behavior University of Minnesota St. Paul Minnesota 55108 USA; ^3^ Department of Biology University of Florida Gainesville Florida 32611 USA

**Keywords:** BiChroM, chromosome number evolution, likelihood function, polyploidy

## Abstract

**Premise of the Study:**

Polyploidy has profound evolutionary consequences for land plants. Despite the availability of large phylogenetic and chromosomal data sets, estimating the rates of polyploidy and chromosomal evolution across the tree of life remains a challenging, computationally complex problem. We introduce the R package chromploid, which allows scientists to perform inference of chromosomal evolution rates across large phylogenetic trees.

**Methods and Results:**

chromploid is an open‐source package in the R environment that calculates the likelihood function of models of chromosome evolution. Models of discrete character evolution can be customized using chromploid. We demonstrate the performance of the BiChroM model, testing for associations between rates of chromosome doubling (as a proxy for polyploidy) and a binary phenotypic character, within chromploid using simulations and empirical data from *Solanum*. In simulations, estimated chromosome‐doubling rates were unbiased and the variance decreased with larger trees, but distinguishing small differences in rates of chromosome doubling, even from large data sets, remains challenging. In the *Solanum* data set, a custom model of chromosome number evolution demonstrated higher rates of chromosome doubling in herbaceous species compared to woody.

**Conclusions:**

chromploid enables researchers to perform robust likelihood‐based inferences using complex models of chromosome number evolution across large phylogenies.

Polyploidy, the acquisition of extra sets of chromosomes, is a recurrent evolutionary process across the plant tree of life (Wendel, 2015). New genomic data have revealed previously undetected ancient polyploidy events (Jiao et al., 2011; Smith et al., 2018), and new mathematical models offer the promise of studying large‐scale macroevolutionary patterns of polyploidy (Mayrose et al., 2010; Glick and Mayrose, 2014; Zenil‐Ferguson et al., 2017; Freyman and Höhna, 2018) by leveraging new data sets of chromosome numbers (Rice et al., 2015) and ploidy values (Bennett and Leicht, 2012), as well as large phylogenies (Smith et al., 2011; Zanne et al., 2014).

Rates of polyploidy in plants may be associated with many phenotypic or life history traits, including sexual system, growth habit, or length of life cycle (see Zenil‐Ferguson et al., 2017). Inferring the rate of polyploidy, and testing its possible links to plant traits, at a macroevolutionary scale requires the development of new phylogenetic comparative methods and software. ChromEvol first addressed rates of chromosome doubling in phylogenies using stochastic models (Mayrose et al., 2010; Glick and Mayrose, 2014). Although rates of chromosome doubling may be used as a proxy for polyploidy, it is not necessarily the same as rates of polyploidy (see Mayrose et al., 2010; Zenil‐Ferguson et al., 2017). ChromEvol, which was developed in a C++ environment, has enabled the estimation of chromosome‐doubling rates in phylogenies with up to 150 taxa (Escudero et al., 2014). More recently, a binary trait and chromosome number evolution model (BiChroM; Zenil‐Ferguson et al., 2017) linked the evolution of chromosome number with the evolution of binary phenotypic traits. The BiChroM implementation, which could evaluate patterns of chromosome evolution in a tree with 4700 taxa, addressed both the computational complexity associated with comparative analyses across phylogenies with thousands of taxa and the need for exponentiation of large and sparse matrices that numerically define the probabilities of chromosome change in discrete trait evolution models (Felsenstein, 1981). In chromosome number transition models, matrices likely have at least a dimension of 25 × 25 (representing at least 25 haploid chromosomes), and their exponential is calculated for every set of parameter values defined in the model. Furthermore, for each set of parameter values, the exponentials have to be calculated for every branch length of the phylogeny. The complexity of the many matrix exponential calculations in large phylogenies is computationally time consuming and numerically unstable (Moler and Van Loan, 2003).

Our challenge was to create a software implementation that would allow for fast and numerically stable probability calculations, resulting in consistent likelihood‐based inferences of rates of chromosome number evolution. The R package chromploid robustly calculates the likelihood function, the probability of observing the chromosome number changes given the phylogenetic tree and a model of chromosome number evolution. The likelihood function contains all the probabilities of chromosome number change along the branches of the phylogeny that are defined via the exponential of large and sparse matrices calculated within chromploid. Furthermore, the likelihood function is key for statistical inferences in both likelihood and Bayesian inference frameworks. The central piece of our software is an R function (chromploid_nloglike), which returns this likelihood function (in negative and logarithmic form for optimization purposes) for parameters that define evolutionary rates of chromosome number or ploidy change. The implementation of the likelihood function in chromploid makes it possible to infer the rate of polyploidy via maximum likelihood estimates (MLEs), likelihood‐confidence intervals, likelihood ratio tests, and profile likelihoods. These statistics allow researchers to assess the evidence and uncertainty of rates of polyploidy across a clade of interest. Currently, chromploid includes three different models of chromosome number evolution: ChromEvolM3 (Mayrose et al., 2010), BiChroM (Zenil‐Ferguson et al., 2017), and a custom model for chromosome number change in *Solanum* L. However, users can easily implement their own discrete character change models, and chromploid can perform the necessary likelihood calculations.

In this study, we demonstrate the performance of chromploid based on three difficult simulation scenarios for BiChroM and we also show one custom application of a model for *Solanum* chromosome number evolution linking chromosome number change to growth form. We performed power analysis simulation for a likelihood ratio test to assess the differences between chromosome‐doubling rates that are linked to a binary trait on trees of different size. The simulations show how different parameter values and numbers of taxa in the phylogeny affect the hypothesis testing framework.

## METHODS AND RESULTS

We evaluated the performance of the chromploid R package (available at https://github.com/roszenil/chromploid) using simulation experiments and an empirical data set. In the simulations, we performed a statistical power analysis of the equal chromosome‐doubling rates hypothesis under the BiChroM model (Zenil‐Ferguson et al., 2017). In the empirical example, we used a custom model to test the link between polyploidy and the woody/herbaceous growth form in *Solanum*.

For the simulations, we used the BiChroM model that estimates the rates of chromosome change associated with two different character states. BiChroM includes 10 parameters that describe the rates of change for gaining one chromosome (*λ*_0_, *λ*_1_), losing one chromosome (*μ*_0_, *μ*_1_), doubling the number of chromosomes (*ρ*_0_, *ρ*_1_), changing the binary character state (*q*_01_, *q*_10_), and changing the binary trait when there are a large number (e.g., >25) of chromosomes (*ε*_0_, *ε*_1_). The numbers 0 or 1 indicate the binary character trait for each taxon in the tree (e.g., 0 = woody; 1 = herbaceous). In the simulation, we fixed the maximum haploid chromosome number to 25, but in chromploid, this value is user defined.

**Figure 1:**
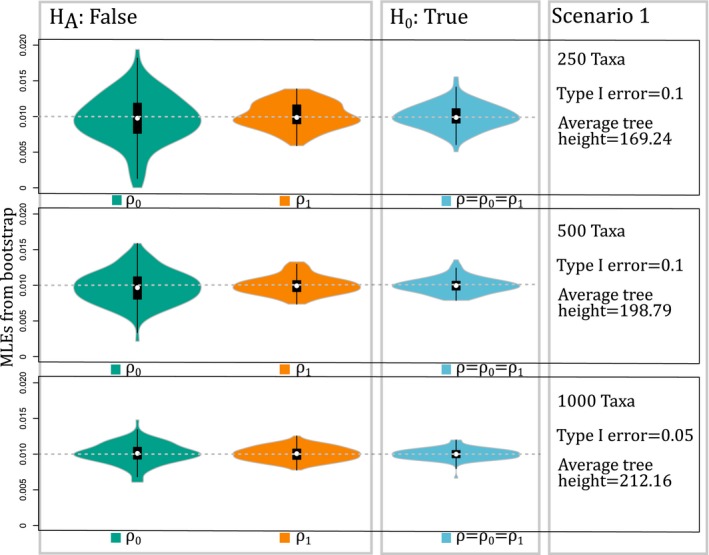
Results of simulations for Scenario 1 using chromploid with the BiChroM model. In Scenario 1, the null hypothesis is true, and it was simulated for trees with 250, 500, and 1000 taxa (shown vertically). An increase in the number of taxa decreases type I error when increasing from 500 to 1000 taxa, and violin plots show a decrease in the variance of the maximum likelihood estimates that are centered at the true value of simulation 0.01 (dotted gray line).

**Figure 2:**
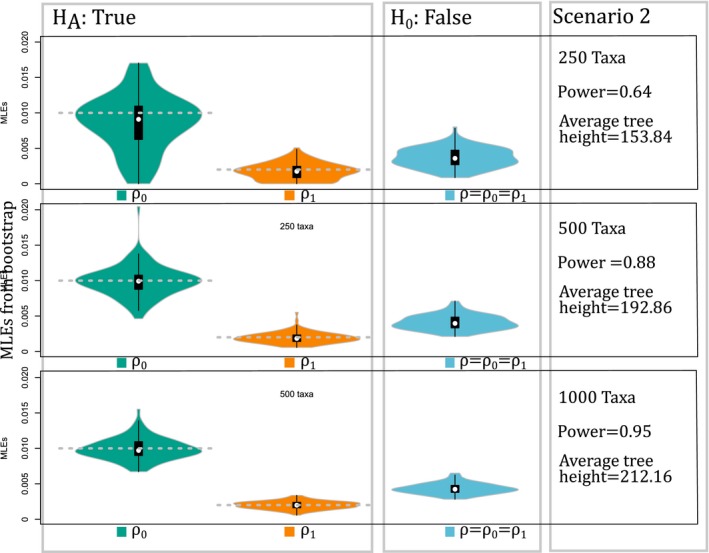
Results of simulations for Scenario 2, where the null hypothesis is false. Simulations show a large power increase by increasing the number of taxa. An increase in the number of taxa also reduces the variance of maximum likelihood estimates, and the difference between the chromosome‐doubling rate estimates becomes larger and closer to the true rate values used in simulations (dotted gray line).

**Figure 3:**
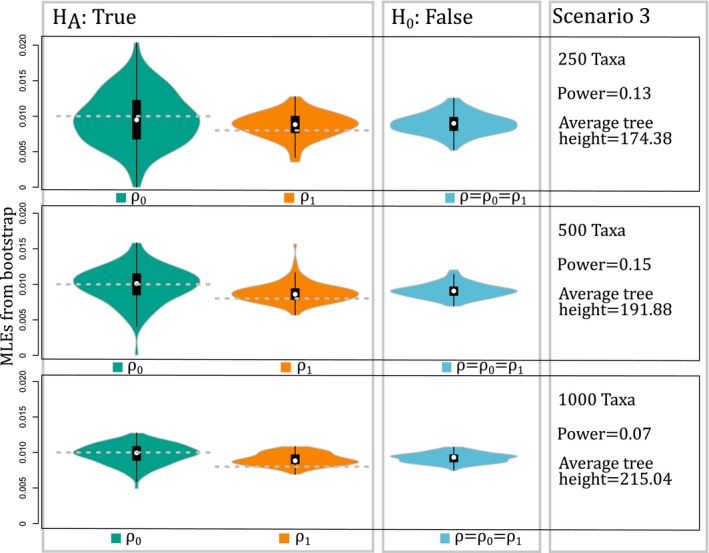
Results of simulations for Scenario 3. Estimates show that the likelihood ratio test has low power, and the power does not increase with more taxa. Increasing taxa reduces the variance of estimates, but not sufficiently for the violin plots to distinguish between the two parameters.

We tested the null hypothesis *H*_0_: *ρ*_0_ = *ρ*_1_ using simulations of three scenarios that were designed to measure type I error and power of the hypothesis *H*_0_. We simulated 300 phylogenetic trees for each scenario with a pure birth process using the R package geiger's function sim.bdtree (Pennell et al., 2014). We generated 100 phylogenetic trees in each scenario with 250 tips (i.e., taxa), 500 tips, and 1000 tips. For each tree, we simulated the chromosome numbers and the value of the binary trait. In the first simulation scenario (*S*_1_), we simulated the BiChroM model using *S*_1_: *ρ*_0_ = *ρ*_1_ = 0.01, meaning that the chromosome‐doubling rates are equal for each binary character state and independent of the binary character trait (i.e., *H*_0_ is true). For the second simulation scenario, we assumed *S*_2_: 0.01 = *ρ*_0_ ≠ *ρ*_1_ = 0.002, meaning that the rates of chromosome doubling are different and depend on the binary trait, and *H*_0_ was false. In the third scenario, *S*_3_: 0.01 = *ρ*_0_ ≠ *ρ*_1_ = 0.008. This last scenario also assumed that *H*_0_ was false, but there was less difference in the value of the rates associated with each binary state. For all three scenarios, the other eight parameters of BiChroM model were fixed at all times in the simulations (*λ*_0_ = 0.01, *λ*_1_ = 0.005, *μ*_0_ = 0.01, *μ*_1_ = 0.005, *q*_01_ = 0.01, *q*_10_ = 0.005, *ɛ*_0_ = 1 × 10^‐6^, *ɛ*_1_ = 1 × 10^‐6^), but they were all estimated in the optimizations. The values fixed for the rest of the parameters were similar in scale to estimates from eudicots (Zenil‐Ferguson et al., 2017). Also, we chose total tree height sizes that yielded zero to four chromosome number changes on average for a single lineage from root to tip (average tree height for simulations is listed in Figs. 1, 2, 3).

The simulated phylogenetic trees and corresponding chromosome number and binary character data sets served as input for the calculation of the negative log‐likelihood of the full BiChroM model (defined via Q_bichrom function), where the maximum likelihood of parameters *ρ*_0_ and *ρ*_1_ were optimized freely, and the reduced BiChroM model (defined via Q_reducedbichrom function), where only the maximum likelihood of one parameter of chromosome doubling *ρ* = *ρ*_0_ = *ρ*_1_ is optimized while the other parameters are optimized freely. The negative log‐likelihood values at the MLEs of the full and reduced BiChroM models (denoted by –*l*) were used to calculate the likelihood ratio test statistic *D* = –2*l*(*reduced BiChroM*) + 2*l*(*full BiChroM*) needed to compare two nested models. This statistic is asymptotically distributed as a chi‐squared distribution with degrees of freedom equal to the difference in the number of estimated parameters (here a $\chi_{\left(1\right)}^{2}$, see Kalbfleisch, 2012). Fixing the significance value of the likelihood ratio test at *α* = 0.05, we estimated type I error in *S*_1_ by calculating the percentage of times that *H*_0_ is (incorrectly) rejected (Fig. 1) and showing the distribution of the MLEs under the full and reduced BiChroM models as violin plots. In *S*_2_ and *S*_3_, we estimated the power of the test by calculating the percentage of times that *H*_0_ is (correctly) rejected (Figs. 2 and 3) and showing the differences of MLEs from the simulation. The original code and results for this example are available at https://github.com/roszenil/bichromRandRB. The R code tutorial for one simulation and likelihood ratio tests in chromploid is described in Appendix S1.

We performed the simulations using 20 processors in the high‐performance computing cluster at the University of Florida; each processor required ≤350 Mb of memory. On average, obtaining the MLEs for trees with 250 tips took 40 min, trees with 500 tips took 90 min, and trees with 1000 tips took <240 min. The simulations in *S*_1_ produced unbiased MLEs, with variance decreasing as the number of taxa in the phylogenetic trees increased (Fig. 1). For these same simulations, the type I error decreased with taxonomic sampling (from 10% to 5%; Fig. 1), showing a 50% decrease in false positives even with a four‐fold increase in the size of the tree. In *S*_2_, the MLEs again were unbiased with decreasing variance as the number of taxa increased (Fig. 2). Furthermore, the violin plots in Fig. 2 showed less overlap as the number of taxa increased. The rate of false negatives (failure to reject *H*_0_; power) with the likelihood ratio test decreased by 24% when increasing from 250 to 500 taxa in the phylogeny (Fig. 2). The results in *S*_3_ again showed that the variance of estimates decreased with an increase in sample size (Fig. 3), but the power of the likelihood ratio test remained small despite the size of the phylogeny (Fig. 3).

We also used chromploid to test if the rates of chromosome number change were linked to the woody (W) or herbaceous (H) growth form in *Solanum* using a custom model. We assembled the input data by first matching the chromosome numbers of the Chromosome Counts Database database (Rice et al., 2015), the growth form data from the Tree of Sex database (Tree of Sex Consortium, 2014), and the Solanaceae‐dated phylogeny from Särkinen et al. (2013). If a taxon could be woody or herbaceous, we coded it as woody, and if there were multiple chromosome numbers recorded for a taxon, we used the largest. The data set included 171 taxa that all had either 12, 18, 24, 36, or 48 chromosomes. We proposed a custom model of chromosome number evolution (defined in a custom Q‐matrix function called Q_solanum) by defining six parameters: (*ρ*_*H*_, *ρ*_*W*_) the chromosome‐doubling rates for herbaceous and woody taxa, respectively; (*ɛ*_*H*_, *ɛ*_*W*_) the 1.5× chromosome increase (i.e., demiploidy) rate for herbaceous and woody taxa, respectively; and (*q*_*HW*_, *q*_*WH*_) the rates of transition between woody and herbaceous states (see graphical model description and Q‐matrix code for chromploid in Appendix S2). We tested the null hypothesis *H*_0_: *ρ*_*H*_ = *ρ*_*W*_ by estimating the reduced model with *ρ* = *ρ*_*H*_ = *ρ*_*W*_ on the same data set and calculating the likelihood ratio test statistic *D* as defined above. We performed the estimations using the same 20 processors in the high‐performance computing cluster at the University of Florida.

On average, obtaining the MLEs for full and reduced models in the *Solanum* data set took 30 min. The likelihood ratio test results in rejection of the null hypothesis *H*_0_ (*D* = 15.655, *P* value = 7.59 × 10^‐5^) that the chromosome‐doubling rate for herbaceous taxa *ρ*_*H*_ is equal to that of woody taxa *ρ*_*W*_. Instead, as in Zenil‐Ferguson et al. (2017), the rates of chromosome doubling are much higher in herbaceous than woody lineages. Based on the amount of difference (~0.13) shown by the MLEs of chromosome‐doubling rates shown in Table 1, the difference in rates of transition between growth forms (*q*_*HW*_ and *q*_*WH*_), and the number of taxa in the matching *Solanum* phylogeny, our confidence in the difference in chromosome‐doubling rates appears to be comparable to the simulations shown in *S*_2_ (Fig. 2).

**Table 1:** Maximum likelihood estimates and likelihood value for full and reduced BiChroM models.^a^

Model/ MLE	*ɛ*_*H*_	*ɛ*_*W*_	*ρ*_*H*_	*ρ*_*W*_	*q*_*HW*_	*q*_*WH*_	Negative log‐likelihood
Full	0.084	1.14 × 10^−19^	0.139	0.0024	0.835	0.453	283.575
Reduced	0.072	2.20 × 10^−17^	0.047		1.12	0.717	291.402

a The likelihood ratio test (*D* =  15.655) rejects the null hypothesis (*P* value = 7.59 × 10^−5^) that the chromosome‐doubling rates *ρ*_*H*_ and *ρ*_*W*_ are equal.

## CONCLUSIONS

Our simulations demonstrate that chromploid provides unbiased estimates of rates of chromosome doubling based on the BiChroM model (Figs. 1, 2, 3), and it easily enables inferences regarding the rates and patterns of chromosomal number evolution on large phylogenetic trees. Variance in the parameter estimates can decrease greatly with increased taxon sampling (Fig. 1), suggesting that chromploid analyses will be most reliable with large data sets, as shown in other comparative models (Davis et al., 2013). In simulations with only 75 taxa, the type I error is 23%, but the power of detecting true differences can be as small as 37% (see Appendix S3). Thus, chromploid likely will only be effective on analyses with at least a few hundred taxa. The simulations also provide reasons to be cautious when interpreting results of chromploid analyses. In Scenario 3 (Fig. 3), there is little power when the difference in chromosome‐doubling rates is small, even when using extremely large phylogenies. Several factors can diminish the power of detecting rate differences. First, when the rates of binary state transitions are unbalanced, it can be especially difficult to estimate parameters linked to binary state with the faster transition rate. This can be observed in Figs. 1, 2, and 3, where violin plots show greater uncertainty for chromosome‐doubling rate associated to state 0 (*ρ*_0_) than to state 1 (*ρ*_1_). Second, the shape of the phylogenetic tree might result in little change in type I or type II error rates when adding additional taxa. Because taxa in the phylogeny do not represent an independent sample, power analyses like the ones implemented here can reveal the importance and effect of the phylogenetic structure on the analyses. A similar idea regarding effective sample size was discussed by Ané (2008) in the context of continuous trait evolution. Third, when chromosome‐doubling rates are very close in value and either the unbalanced binary trait transitions and/or the small effective sample size problems appear, the variances of estimates may not decrease fast enough to result in a significant likelihood ratio test (Fig. 3).

An effective tool for examining patterns of chromosome number evolution across large phylogenies ideally will enable the user to create custom models and then allow for the calculation and optimization of the likelihood function. As we demonstrated with our *Solanum* example, scientists can use chromploid to customize models of chromosome evolution and perform robust statistical inferences using the models in the R environment. The likelihood function requires a calculation of probabilities from large and sparse matrices where a set of parameter values has to be evaluated for every single branch of the phylogenetic tree, greatly increasing the complexity in the calculation of the likelihood function (Felsenstein, 1981). Therefore, chromploid's main numerical challenge was to obtain quick calculations of the likelihood function in phylogenetic trees with hundreds, if not thousands, of taxa and with many possible chromosome numbers (or discrete character states). The fast calculation of likelihood functions for chromosome number evolution models in chromploid allows users to perform four key statistical inferential tasks: (1) power analyses for detection of minimum sample sizes and hypothesis testing robustness, (2) exploration of difficult likelihood surfaces common to phylogenetic context (Chor et al., 2000), (3) calculation of confidence intervals and relative profile likelihoods for parameters of interest (Zenil‐Ferguson et al., 2017), and (4) assessment of parameter estimability (Ponciano et al., 2012).

Implementing chromosomal and ploidy evolution models in the R environment in chromploid allows users to leverage other tools for phylogenetic analyses, like ancestral state reconstruction using diversitree (FitzJohn, 2012) or phylomap (Irvahn and Minin, 2014), and to visualize character evolution across the tree using phytools (Revell, 2012). In the future, it will be straightforward to implement more sophisticated or user‐customized models in chromploid. Future models can include heterogeneity in the patterns of chromosomal evolution across the tree or uncertainty in the chromosome numbers at the tips. Also, Bayesian inferences using these models may be implemented in other platforms like RevBayes (Höhna et al., 2016). A first implementation for ChromEvol and BiChroM models that can be used on small phylogenies has been made in RevBayes (Freyman and Höhna, 2018).

## DATA ACCESSIBILITY

The chromploid R package and tutorials can be found at https://github.com/roszenil/chromploid. Files with results from simulations for the three scenarios, the *Solanum* data set and optimization, and code used for this article can be found at https://github.com/roszenil/bichromRandRB.

## References

[aps31037-bib-0001] Ané, C. 2008 Analysis of comparative data with hierarchical autocorrelation. Annals of Applied Statistics 2: 1078–1102.

[aps31037-bib-0002] Bennett, M. D. , and I. J. Leicht . 2012 Royal Botanic Gardens, Kew: Plant DNA C‐values database (release 6.0). Available at: http://data.kew.org/cvalues/ [accessed 7 October 2017].

[aps31037-bib-0003] Chor, B. , M. D. Hendy , B. R. Holland , and D. Penny . 2000 Multiple maxima of likelihood in phylogenetic trees: An analytic approach. Molecular Biology and Evolution 17: 1529–1541. 11018159 10.1093/oxfordjournals.molbev.a026252

[aps31037-bib-0004] Davis, M. P. , P. E. Midford , and W. Maddison . 2013 Exploring power and parameter estimation of the BiSSE method for analyzing species diversification. BMC Evolutionary Biology 13: 38. 23398853 10.1186/1471-2148-13-38 PMC3583807

[aps31037-bib-0005] Escudero, M. , S. Martín‐Bravo , I. Mayrose , M. Fernández‐Mazuecos , O. Fiz‐Palacios , A. L. Hipp , M. Pimentel , et al. 2014 Karyotypic changes through dysploidy persist longer over evolutionary time than polyploid changes. Plos One 9: e85266. 24416374 10.1371/journal.pone.0085266 PMC3887030

[aps31037-bib-0006] Felsenstein, J. 1981 Evolutionary trees from DNA sequences: A maximum likelihood approach. Journal of Molecular Evolution 17: 368–376. 7288891 10.1007/BF01734359

[aps31037-bib-0007] FitzJohn, R. G. 2012 Diversitree: Comparative phylogenetic analyses of diversification in R. Methods in Ecology and Evolution 3: 1084–1092.

[aps31037-bib-0008] Freyman, W. A. , and S. Höhna . 2018 Cladogenetic and anagenetic models of chromosome number evolution: A Bayesian model averaging approach. Systematic Biology. 67: 195–215. 28945917 10.1093/sysbio/syx065

[aps31037-bib-0009] Glick, L. , and I. Mayrose . 2014 ChromEvol: Assessing the pattern of chromosome number evolution and the inference of polyploidy along a phylogeny. Molecular Biology and Evolution 31: 1914–1922. 24710517 10.1093/molbev/msu122

[aps31037-bib-0010] Höhna, S. , M. J. Landis , T. A. Heath , B. Boussau , N. Lartillot , B. R. Moore , J. P. Huelsenbeck , and F. Ronquist . 2016 RevBayes: Bayesian phylogenetic inference using graphical models and an interactive model‐specification language. Systematic Biology 65: 726–736. 27235697 10.1093/sysbio/syw021 PMC4911942

[aps31037-bib-0011] Irvahn, J. , and V. N. Minin . 2014 Phylogenetic stochastic mapping without matrix exponentiation. Journal of Computational Biology 21: 676–690. 24918812 10.1089/cmb.2014.0062 PMC4148059

[aps31037-bib-0012] Jiao, Y. , N. J. Wickett , S. Ayyampalayam , A. S. Chanderbali , L. Landherr , P. E. Ralph , L. P. Tomsho , et al. 2011 Ancestral polyploidy in seed plants and angiosperms. Nature 473: 97–100. 21478875 10.1038/nature09916

[aps31037-bib-0013] Kalbfleisch, J. G. 2012 Probability and statistical inference. Springer Science & Business Media, New York, New York, USA.

[aps31037-bib-0014] Mayrose, I. , M. S. Barker , and S. P. Otto . 2010 Probabilistic models of chromosome number evolution and the inference of polyploidy. Systematic Biology 59: 132–144. 20525626 10.1093/sysbio/syp083

[aps31037-bib-0015] Moler, C. , and C. Van Loan . 2003 Nineteen dubious ways to compute the exponential of a matrix, twenty‐five years later. SIAM Review 45: 3–49.

[aps31037-bib-0016] Pennell, M. W. , J. M. Eastman , G. J. Slater , J. W. Brown , J. C. Uyeda , R. G. FitzJohn , M. E. Alfaro , and L. J. Harmon . 2014 geiger v2.0: An expanded suite of methods for fitting macroevolutionary models to phylogenetic trees. Bioinformatics 30: 2216–2218. 24728855 10.1093/bioinformatics/btu181

[aps31037-bib-0017] Ponciano, J. M. , J. G. Burleigh , E. L. Braun , and M. L. Taper . 2012 Assessing parameter identifiability in phylogenetic models using data cloning. Systematic Biology 61: 955–972. 22649181 10.1093/sysbio/sys055 PMC3478565

[aps31037-bib-0018] Revell, L. J. 2012 phytools: An R package for phylogenetic comparative biology (and other things). Methods in Ecology and Evolution 3: 217–223.

[aps31037-bib-0019] Rice, A. , L. Glick , S. Abadi , M. Einhorn , N. M. Kopelman , A. Salman‐Minkov , J. Mayzel , et al. 2015 The Chromosome Counts Database (CCDB): A community resource of plant chromosome numbers. New Phytologist 206: 19–26. 25423910 10.1111/nph.13191

[aps31037-bib-0020] Särkinen, T. , L. Bohs , R. G. Olmstead , and S. Knapp . 2013 A phylogenetic framework for evolutionary study of the nightshades (Solanaceae): A dated 1000‐tip tree. BMC Evolutionary Biology 13: 214. 24283922 10.1186/1471-2148-13-214 PMC3850475

[aps31037-bib-0021] Smith, S. A. , J. M. Beaulieu , A. Stamatakis , and M. J. Donoghue . 2011 Understanding angiosperm diversification using small and large phylogenetic trees. American Journal of Botany 98: 404–414. 21613134 10.3732/ajb.1000481

[aps31037-bib-0022] Smith, S. A. , J. W. Brown , Y. Yang , R. Bruenn , C. P. Drummond , S. F. Brockington , J. F. Walker , et al. 2018 Disparity, diversity, and duplications in the Caryophyllales. New Phytologist 217: 836–854. 28892163 10.1111/nph.14772

[aps31037-bib-0023] Tree of Sex Consortium . 2014 Tree of Sex: A database of sexual systems. Scientific Data 1: 140015. 25977773 10.1038/sdata.2014.15 PMC4322564

[aps31037-bib-0024] Wendel, J. F. 2015 The wondrous cycles of polyploidy in plants. American Journal of Botany 102: 1753–1756. 26451037 10.3732/ajb.1500320

[aps31037-bib-0025] Zanne, A. E. , D. C. Tank , W. K. Cornwell , J. M. Eastman , S. A. Smith , R. G. FitzJohn , D. J. McGlinn , et al. 2014 Three keys to the radiation of angiosperms into freezing environments. Nature 506: 89–92. 24362564 10.1038/nature12872

[aps31037-bib-0026] Zenil‐Ferguson, R. , J. M. Ponciano , and J. G. Burleigh . 2017 Testing the association of phenotypes with polyploidy: An example using herbaceous and woody eudicots. Evolution 71: 1138–1148. 28295270 10.1111/evo.13226

