# Supplementary material for: chromploid: An R package for chromosome number evolution across the plant tree of life
Source: Appl Plant Sci. 2018 Apr 11;6(3):e1037. doi: 10.1002/aps3.1037 (PMC5895187; doi:10.1002/aps3.1037)
Supplement: Supplementary file 1 [file APS3-6-e1037-s001.docx]

Zenil-Ferguson et al.—Applications in Plant Sciences 2018 6(3)—Data Supplement S1.

DOI: 10.1002/aps3.1037

**Appendix S1.** Code to simulate a BiChroM under scenario 2.

Libraries needed for the simulation example

library("geiger")
library("chromploid")

## Equal chromosome number doubling rate in BiChroM simulation

First, choose some parameter values in log scale for the 10 parameters of BiChroM and the maximum number of haploid chromosomes to build the Q-matrix.

# l.0, l.1, m.0, m.1, r.0, r.1, prob.01, prob.10, e.0, e.1
# input in log-scale
params <- log(c(0.01, 0.005, 0.01, 0.005, 0.01, 0.01, 0.01, 0.005,
 1e-06, 1e-06))
# Maximum number of haploid chromosomes
N <- 25

Then, simulate a phylogeny with 250 tips and BiChroM process on top of it

# simulating a phylogeny with 250 tips (birth process)
tree <- sim.bdtree(b = 0.03, stop = "taxa", n = 250) # Approximatedly 110 million years

# Bichrom.model defined via Q-matrix
bichrom.model <- Q_bichrom(log.theta = params, size = N)
# Simulating chromosome number and binary trait change
# states, root 27 reflects that the simulation starts at the
# root having binary trait 1 with 1 haploid chromosome number
res <- chromploid_sim.char(phy = tree, par = bichrom.model, nsim = 1,
 root = 27)

Now you have some data.

## Optimization of full and reduced models of BiChroM

The optimization process requires you to assign a probability distribution at the root of the tree. In this example, the distribution is uniform (we really don't know the chromosome number or binary trait at the root). Then, you can use a wrapper function like bichrom_optim to optimize full and reduced BiChroM models with the data you just generated. If you are interested in optimizing your own model you can just follow what is inside this function but with your own custom Q-matrix (see *Solanum* example).

## Uniform distribution of values at the root
p.0 <- rep(1, 2 * (N + 1))/(2 * (N + 1))
model.args <- list(size = 25)
optimization.bichrom <- bichrom_optim(phy.tree = tree, tip.values = res,
 model = "bichrom", model.args = model.args, starting.val = params,
 root.dist = p.0)

model.args = list(size = 50, equal.param = c("rho0", "rho1"),
 location.par = 5)

optimization.reducedbichrom <- bichrom_optim(phy.tree = tree,
 tip.values = res, model = "reducedbichrom", model.args = model.args,
 startin.val = params[-6], root.dist = p.0)

## Likelihood ratio test

As you have already calculated the negative log-likelihoods using bichrom_optim function, you only need to calculate statistic D and the *P* value as follows:

neglog.red<-optimization.reducedbichrom$nloglike
neglog.full<-optimization.bichrom$nloglike
alpha<-0.05
D =2*(neglog.red-neglog.full)
p.value<-pchisq(D, lower.tail=FALSE, df=1)
 if(p.value<0.05){
 reject<-0
 }else{reject<-1}
``
