# Supplementary material for: chromploid: An R package for chromosome number evolution across the plant tree of life
Source: Appl Plant Sci. 2018 Apr 11;6(3):e1037. doi: 10.1002/aps3.1037 (PMC5895187; doi:10.1002/aps3.1037)

Appendix S2. Graphical representation of the model proposed to analyze the *Solanum* data set. The *Solanum* model is a continuous time Markov Chain (Mkn model) containing 10 states, five herbaceous and five woody, each with chromosome number values 12, 18, 24, 36, and 48. It is defined via six parameters: two for chromosome doubling as a proxy of polyploidy (ρ_H, ρ_w), two for increase by six or 12 chromosomes as a proxy of demiploidy (ε_H, ε_W), and two representing the transitions between woody and herbaceous states (q_{HW}, q_{WH}).

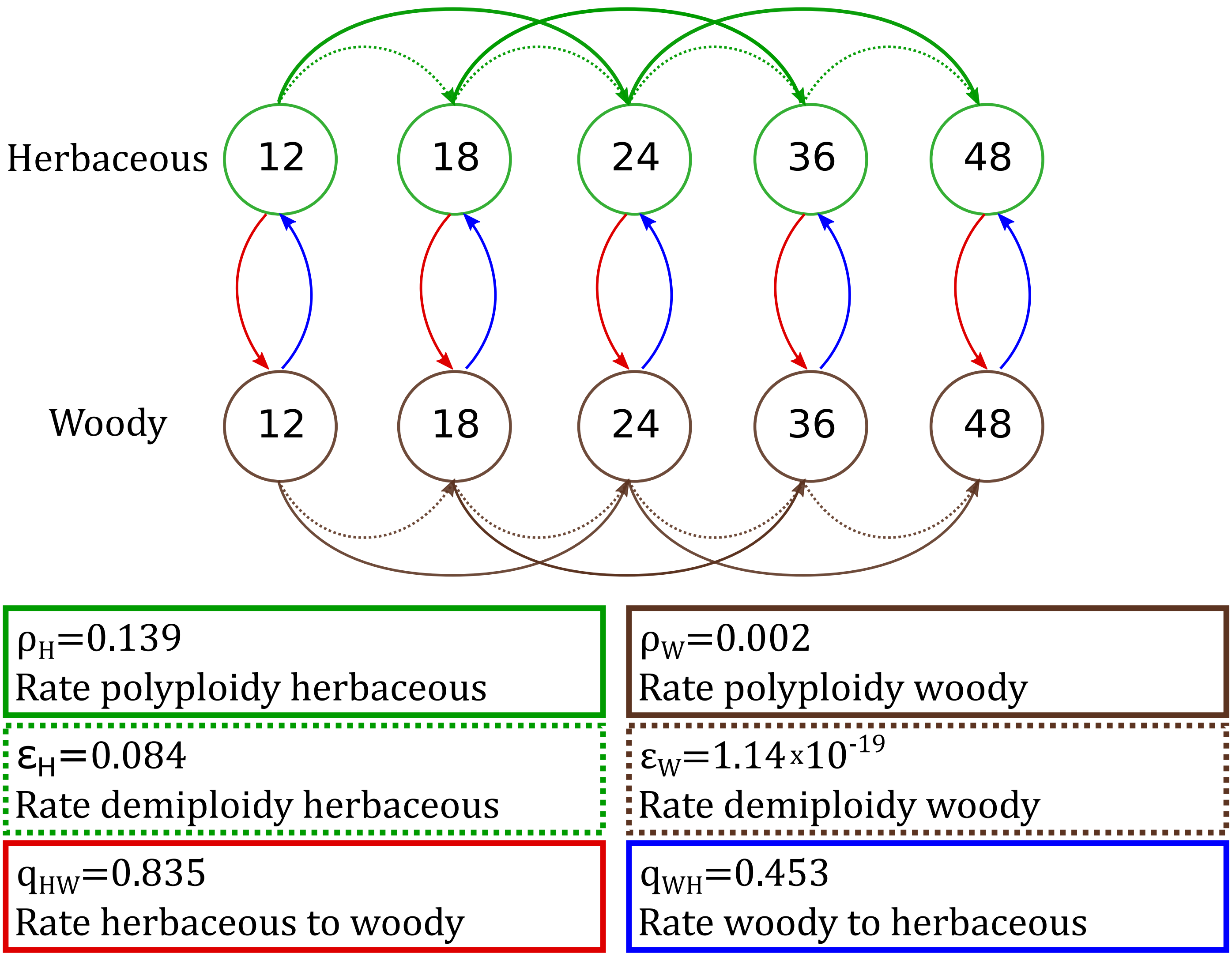

Supplement: Supplementary file 2 [file APS3-6-e1037-s002.pdf]
