# Supplementary material for: chromploid: An R package for chromosome number evolution across the plant tree of life
Source: Appl Plant Sci. 2018 Apr 11;6(3):e1037. doi: 10.1002/aps3.1037 (PMC5895187; doi:10.1002/aps3.1037)

Zenil-Ferguson et al.—Applications in Plant Sciences 2018 6(3)—Data Supplement S3.
DOI

Appendix S3. Results of simulations for trees with 75 taxa under scenarios 1 and 2. Although type I error is 23%, the power of detection for a large difference in polyploidy rates remains small, showing the need of increasing sample size.

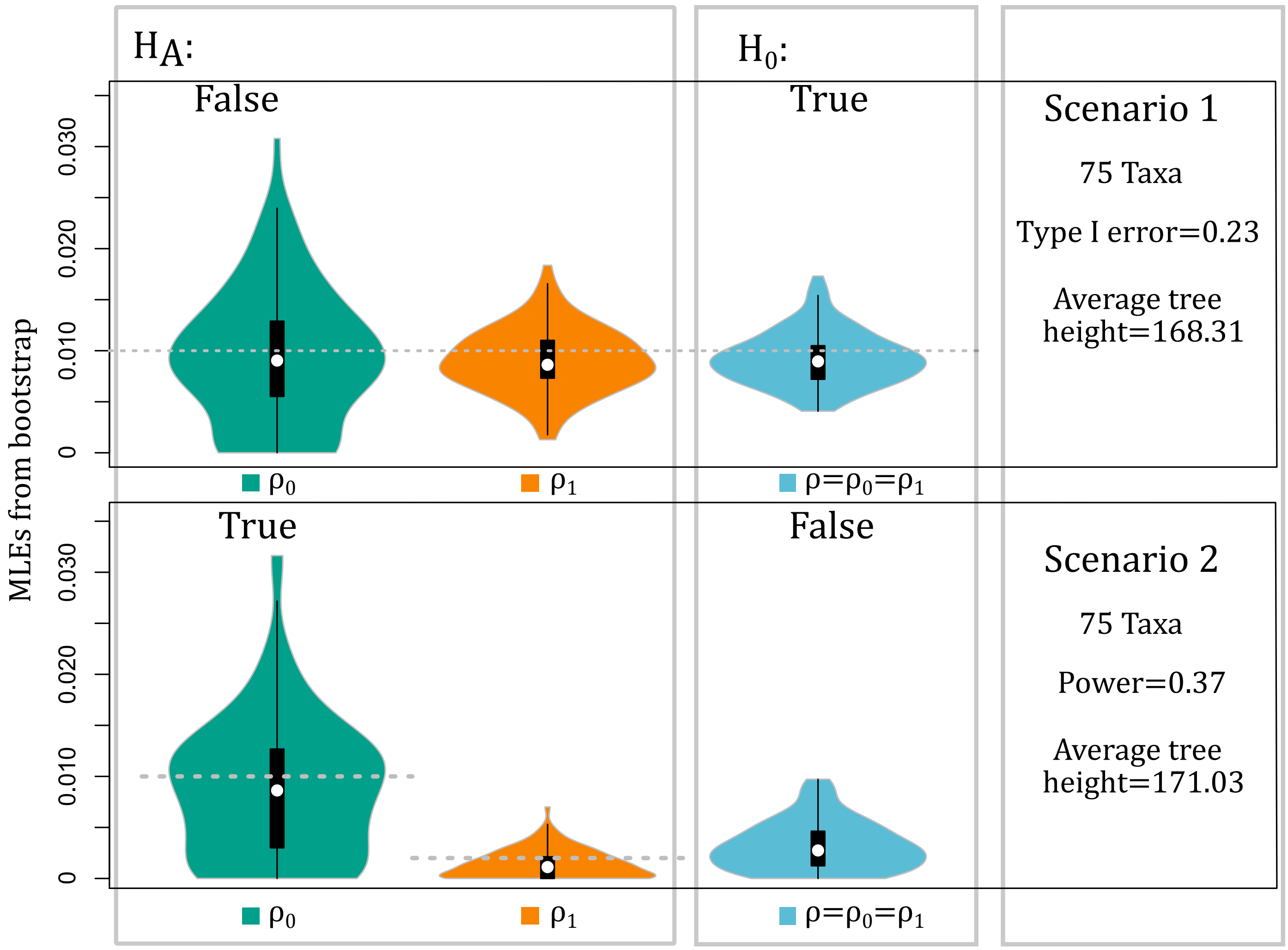

Supplement: Supplementary file 3 [file APS3-6-e1037-s003.pdf]
